# Tunneled dialysis catheter placement in intensive care unit patients: influence of catheter tip design on clinical performance

**DOI:** 10.1186/s42155-025-00596-1

**Published:** 2025-09-29

**Authors:** Alexey Gurevich, Gregory J. Nadolski, Ansar Z. Vance, Ryan-Assaad El-Ghazal, Raphael Cohen, Timothy W. I. Clark, Matthew L. Hung

**Affiliations:** 1 Division of Interventional Radiology, Department of Radiology, University of Pennsylvania, Philadelphia, PA USA; 2 Department of Radiology, Cleveland Clinic, Cleveland, OH USA; 3 Division of Nephrology, Department of Medicine, University of Pennsylvania, Philadelphia, PA USA; 4 Division of Interventional Radiology, Department of Radiology, Stanford University School of Medicine, 300 Pasteur Dr., Stanford, CA 94304 USA

**Keywords:** Tunneled Dialysis Catheter, Venous Access, Ash-Split, VectorFlow, Catheter Malfunction

## Abstract

**Purpose:**

This study evaluates catheter failure rates between a helical-tip catheter and a traditional split-tip catheter among intensive care unit (ICU) patients undergoing tunneled dialysis catheter (TDC) placement.

**Materials and methods:**

We analyzed 1734 TDCs placed over seven years in a retrospective fashion, focusing on 340 catheters used in an ICU setting. Of these, 112 were VectorFlow catheters (32.9%), and 228 were Ash-Split catheters (67.1%). Catheter failure rates due to malfunction or infection were assessed using Kaplan–Meier analysis, while contributing factors were evaluated using Cox proportional hazards modeling.

**Results:**

Within 90 days, 34.8% of patients experienced catheter failure. The VectorFlow catheter demonstrated superior unassisted patency at 30, 60, 90, and 180 days compared to the Ash-Split catheter (87.4 ± 3.6%, 78.0 ± 5.1%, 75.1 ± 5.0% and 60.1 ± 8.2% compared with 75.0 ± 3.3%, 62.8 ± 4.1%, 60.7 ± 4.2% and 44.3 ± 5.5% respectively, *P* = 0.022). Adjusted hazard ratios indicated Ash-Split catheters were nearly twice as likely to fail according to both univariate (1.72, *P* = 0.024) and multivariate (1.89, *P* = 0.017) modeling.

**Conclusion:**

The findings suggest that the VectorFlow catheter demonstrates significantly better primary unassisted patency over the Ash-Split design in ICU settings, supporting its preferential use.

## Introduction

Tunneled dialysis catheters (TDCs) are an essential form of vascular access for managing acute and chronic renal failure among intensive care unit (ICU) patients. Escalating utilization of renal replacement therapy, necessitated by increasing national rates of renal impairment, underscores the importance of optimizing vascular access options [1]. Over 700,000 Americans require renal replacement therapy, with over 83% of patients initiating access with a TDC [2]. Despite limitations of thrombosis, infection and central venous stenosis, TDCs not only serve as a bridge to more permanent solutions in acute settings but also provide a means for dialysis when other modes of permanent access fail, highlighting the indispensability of TDCs in the continuum of renal care [3].

The dynamics within the ICU setting, characterized by patients in critical condition with compromised intravascular volumes and heightened acuity, further highlight the need for optimizing TDC performance. An estimated 10–15% of ICU patients require renal replacement therapy, and differences in the longevity and reliability of TDCs can markedly influence patient outcomes and healthcare costs [4, 5]. TDC outcomes within the ICU setting have not been specifically studied, leaving a gap in evidence-based practice for this vulnerable population.

Recent advancements in catheter design, including the development of a symmetrical helical-tip VectorFlow catheter (Teleflex, Wayne, Pennsylvania), have furthered insight regarding catheter performance, specifically in terms of primary unassisted patency and failure rates attributable to malfunction and infection [6, 7]. Symmetrical tip catheters tend to have ease of atrial positioning as compared to step-tip and split-tip designs, and exhibit reduced recirculation during reversal of the arterial and venous lumens during dialysis [8, 9]. VectorFlow specifically creates spiral laminar flow through helical interfaces at the catheter tip and thereby reduces catheter thrombogenicity and recirculation [10, 11].

Previous retrospective data showed the VectorFlow catheter to have improved intervention-free patency compared with a split-tip catheter [6]. In addition, a prospective study showed high sustained Kt/V values [12] and a randomized controlled trial showed noninferiority compared to a conventional symmetric-tip catheter with consistently better dialysis adequacy through higher Kt/V [7]. This retrospective study focuses on a 7-year experience with the VectorFlow catheter compared to a split-tip device, the Ash-Split Cath catheter (Medcomp, Harleysville, Pennsylvania), in the ICU setting.

## Materials and methods

This retrospective study was approved by the local institutional review board and is in compliance with the Health Insurance Portability and Accountability Act (HIPAA). A prospectively maintained information system (Hi-IQ; ConexSys, Lincoln, Rhode Island) was searched between 2015 and 2022 for patients who underwent placement of a de novo TDC while in the ICU setting. These patients received either the symmetric tip Arrow–Clark VectorFlow catheter or the conventional split-tip Ash-Split Cath at the discretion of the operating interventional radiologist; both catheters were placed throughout the duration of the study period.

Procedures were performed at two academic tertiary care centers by experienced, fellowship-trained radiologists. All catheters were inserted via a right or left internal jugular vein, chosen based on venous patency and operator preference, under ultrasound and fluoroscopic guidance. Translumbar or transfemoral placements were excluded. Skin preparation involved 2% chlorhexidine unless contraindicated, in which case betadine was used. Catheters were advanced using peel-away sheaths with aerostatic valves and positioned per manufacturer guidelines: in the mid-right atrium for VectorFlow and the middle-to-lower right atrium for both tips of the Ash-Split. Operators reviewed fluoroscopic landmarks and pre-procedural imaging to ensure the catheter tip was appropriately positioned. Venotomy sites were closed with absorbable sutures (4.0 polyglactin (Vicryl, Ethicon, Raritan, NJ) and/or tissue adhesive (Dermabond, Ethicon). Post-procedurally, catheters were locked with heparin (100 U/mL) or sealed with needleless barrier caps after a flush with heparinized saline (Tego cap; ICU Medical, San Clemente, California) per operator’s choice.

The primary outcome was defined using Society of Interventional Radiology guidelines for primary unassisted catheter patency [13]; catheter failure was determined by instances of infection, thrombosis, or both requiring catheter removal or over-the-wire exchanges since initial placement. Catheter infection was determined on a clinical basis, either through local signs of infection (e.g. catheter or tunnel site erythema or purulence) or bacteremia/fungemia not attributable to another source. Catheter patency was censored at death or catheter removal for renal recovery or functioning permanent access. Catheter placement indication was categorized as either de-novo initiation of dialysis or failed surgical access.

Statistical analyses employed Fisher’s exact test for dichotomous variables and t-tests or Mann–Whitney tests for continuous variables, depending on data distribution. To account for multiple hospitals, clinical comorbidities, and variability of dialysis indication in the ICU, the Acute Physiology and Chronic Health Evaluation II (APACHE II) score was calculated at the time of catheter insertion [14], and intergroup comparison was performed with the Mann–Whitney test. Rates of catheter failure requiring guidewire exchange or removal from a composite outcome of malfunction or infection were compared with Kaplan Meier estimates. Cox proportional hazards models assessing covariates such as catheter type (VectorFlow vs. Ash-Split Cath), side of insertion (left or right), age, sex, body mass index (BMI), catheter length (tip to cuff), and indication. Statistical significance was set at P < 0.05, with all analyses performed using Prism software (Version 9.4.1, GraphPad, San Diego, California).

## Results

### Patient characteristics

During a 7-year period from 2015 to 2022, a total of 340 de novo tunneled dialysis catheters (228 Ash-Split catheters, 112 VectorFlow catheters) were placed in ICU patients who required initiation of hemodialysis due to renal failure or failed surgical access (e.g. non-maturating or thrombosed arteriovenous fistula or graft, failed peritoneal dialysis). All procedures included in this study were performed without immediate complications.

Patient demographics, clinical comorbidities, and catheter configurations are listed in Table 1. There were no significant differences in age, sex, prevalence of hypertension or diabetes, TDC indication or APACHE II scores between the two catheter groups. Of the patients who had an APACHE II score available, 84% presented with acute renal failure. BMI was significantly elevated in the VectorFlow group (31.8 ± 9.0 kg/m2 vs 29.4 ± 8.6 kg/m2, P = 0.012). The Ash-Split group had a higher percentage of left-sided catheters (23.2% vs 12.5%, P = 0.02), and a slightly longer mean catheter length as compared to the VectorFlow group (23.5 ± 2.5 cm vs 22.9 ± 2.2 cm, P = 0.005).

**Table 1:** Baseline Clinical Characteristics

	**VectorFlow (n = 112)**	**Ash Split Cath (n = 228)**	**P Value**
Sex (M/F)	57/55	140/88	0.079^†^
Mean Age (y)	59.6 ± 15.4	61.4 ± 13.3	0.402^‡^
BMI (kg/m^2^)	31.8 ± 9.0	29.4 ± 8.6	**0.012**^‡^
Diabetes (Yes/No)	54/58	110/118	0.999^†^
Hypertension (Yes/No)	91/21	177/51	0.482^†^
Placement Side (L/R)	14/98	53/175	**0.020**^†^
Catheter Length (cm)	22.9 ± 2.2	23.5 ± 2.5	**0.005**^‡^
Indication (De-novo/Surgical failure)	106/6	221/7	0.368^†^
Mean APACHE II Score	24.3 ± 6.1 (n = 78)	25.6 ± 6.1 (n = 88)	0.165^§^

Data presented as mean ± SD as appropriate

*BMI* body mass index, *APACHEII* acute physiology and chronic health evaluation

^†^ = Fisher exact test; ‡ = Mann–Whitney test; § = Student’s *t* test

### Catheter performance

**Fig. 1:**
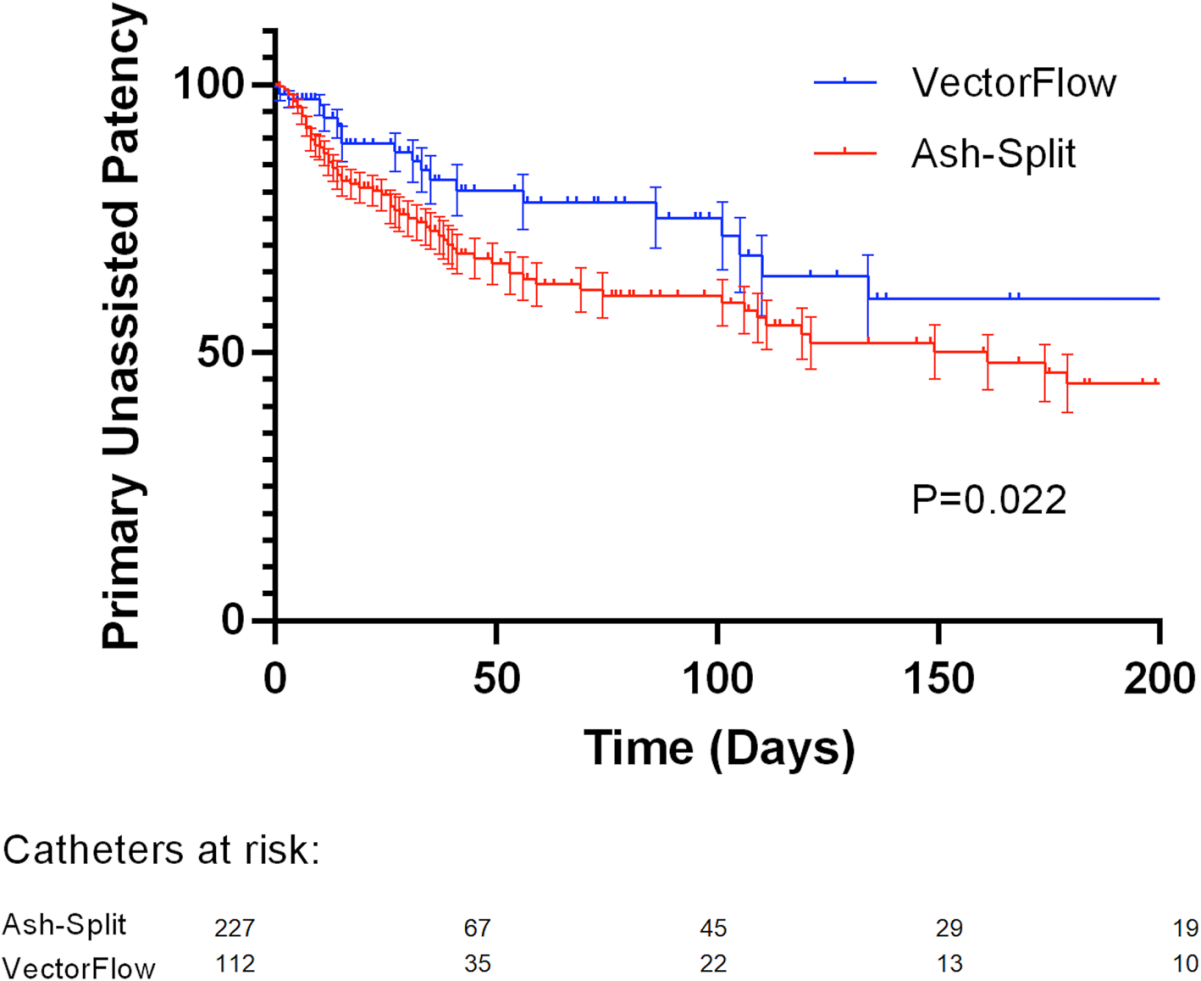
Kaplan–Meier estimate of de novo catheter patency. Catheter primary unassisted patency, defined as freedom from infection or malfunction requiring catheter exchange or removal, was significantly improved with the VectorFlow catheter (n = 112) compared to the Ash-Split Cath (n = 228, P = 0.022)

Within 90 days, 34.8% of ICU placed catheters failed. Primary unassisted patency at 30, 60, 90, and 180 days was significantly higher for VectorFlow catheters: 87.4% ± 3.6%, 78.0% ± 5.1%, 75.1% ± 5.0%, and 60.1% ± 8.2% versus 75.0% ± 3.3%, 62.8% ± 4.1%, 60.7% ± 4.2%, and 44.3% ± 5.5% for Ash-Split (Univariate HR of 0.59, 95% CI of 0.39–0.89, P = 0.022 by log-rank test, Fig. 1). Cumulative catheter-days were 14,840 days in the Ash-Split Cath catheter group and 7678 days in the VectorFlow catheter group.

Catheter removal or exchange for a suspected infection occurred in 11 patients with VectorFlow and 30 patients with Ash-Split Cath. The rate of infections among patients with VectorFlow catheters was 0.14 per 100 catheter-days. The rate of infections among patients with Ash-Split Cath catheters was 0.20 per 100 catheter-days. This observed difference in infection events between the two catheter types did not reach statistical significance (P = 0.48). Catheter removal or exchange for a malfunction occurred in 12 patients with VectorFlow and 49 patients with Ash-Split Cath. The rate of malfunction among patients with VectorFlow catheters was 0.16 per 100 catheter-days. The rate of malfunction among patients with Ash-Split Cath catheters was 0.33 per 100 catheter-days. This observed difference in malfunction events between the two catheter types was statistically significant (P = 0.016). The primary composite endpoint of catheter removal or exchange due to infection or malfunction occurred in 23 patients with VectorFlow for a rate of 0.29 per 100 catheter-days, and in 79 patients with Ash-Split Cath for a rate of 0.53 per 100 catheter days. This observed difference in total events was statistically significant (P = 0.008).

Multivariate stepwise Cox proportional-hazards modeling was used to estimate the effect of clinical predictors on catheter failure and to control for confounding variables (Table 2). The VectorFlow catheter hazard ratio was 0.53 (95% CI 0.32–0.84, P = 0.009), indicating the Ash-Split Cath was 1.9 times more likely to fail. BMI also had a significant result on the primary outcome (HR of 1.02, 95% CI of 1.00–1.05, P = 0.045). Age, sex, indication for placement, laterality, and catheter length were not associated with increased risk of catheter failure in this ICU patient population. Subset analysis of right-sided catheters revealed a VectorFlow hazard ratio of 0.46 (95% CI of 0.25–0.78, P = 0.006), and analysis of left-sided catheters revealed a VectorFlow hazard ratio of 0.96 (95% CI of 0.29 to 2.66, P = 0.94).

**Table 2:** Multivariate Cox Proportional Hazards Modeling of Catheter Failure

Variable	HR	95% CI	P Value
Age	1.00	0.99–1.01	0.94
Sex	1.09	0.71–1.69	0.70
VectorFlow (vs. Ash-Split Cath)	0.53	0.32–0.84	**0.009**
BMI	1.02	1.00–1.05	**0.045**
Indication for catheter placement	1.26	0.48–2.75	0.59
Left jugular placement (vs right)	1.29	0.72–2.18	0.37
Catheter Length (cm)	0.92	0.84–1.04	0.14

*BMI* body mass index, *CI* confidence interval, *HR* hazard ratio

## Discussion

The use of TDCs within intensive care settings is critical in managing both acute and chronic renal failure among ICU patients. With the rising incidence of renal impairment and the subsequent demand for renal replacement therapy, the selection of an optimal vascular access method has emerged as a significant concern. This necessity is underscored by the reliance on TDCs by a substantial number of patients undergoing hemodialysis, requiring a vascular access that not only facilitates effective dialysis but also minimizes the risk of complications [15]. In the ICU setting, patients have increased fluid and electrolyte disturbances, higher rates of renal replacement therapy initiation, and worse overall outcomes compared to floor or outpatient populations initiating hemodialysis [4].

The VectorFlow catheter has helical transition zones which impart spiral laminar flow to blood entering and leaving the distal lumens [10, 11]. Helical flow has been shown to reduce platelet adhesion [16] and thrombus formation when imparted to the design of a variety of cardiovascular devices including arteriovenous grafts [17], inferior vena cava filters [18], and mechanical circulatory support devices [19]. Unlike the Ash Split catheter, the VectorFlow catheter also does not taper towards the end hole, allowing for reduced flow friction. Primary unassisted TDC patency was superior with the VectorFlow catheter as compared to the Ash Split catheter at multiple timepoints throughout the first 180 days. The 90-day primary patency rate of 75.1% for VectorFlow in the present study is mildly reduced compared to its patency in the randomized clinical trial studying its performance, but the randomized study did not limit patient selection to ICU patients [7]. Despite being placed in patients with significantly greater BMI, a known risk factor for catheter malfunction [4], the VectorFlow still had significantly higher primary patency than the Ash-Split Cath. Catheter laterality and length were not significantly impactful in this study’s patient population.

Examination of individual instances of catheter removal or exchanges due to infection or malfunction showed significantly improved rates for VectorFlow at 0.29 per 100 catheter-days, as compared to 0.53 per 100 catheter-days for the Ash-Split Cath, suggesting that catheter tip design may account for the differences in clinical outcomes observed in this study.

This study has several limitations. This retrospective analysis was performed across 2 hospitals at a single tertiary academic institution with each catheter predominating at their respective hospitals due to operator preference. Although catheters were placed in multiple hospitals, they both belong to the same academic institution, managed by the same renal dialysis teams. In order to assess patient acuity at the time of TDC placement, APACHE II scores were calculated to grade the severity of illness in the ICU setting; APACHE II scores were not significantly different, indicating similar disease severity between the two groups. Although catheter tips were optimally positioned in the fluoroscopy suite, tip position was not routinely re-assessed on a subsequent upright chest x-ray. Due to the retrospective nature of the study, it is difficult to discern whether catheters removed for infection were due to true catheter-associated infection, infection from another source, or undifferentiated sepsis. However, there was still a significant difference in catheter patency between the two groups when examining malfunction alone, which is an objective event, and a less ambiguous outcome compared to infection. Additionally, the presence of co-indwelling central venous catheters in the ICU setting was not assessed; this may have affected the available sites for TDC placement but likely has minimal impact on TDC function. Due to the retrospective nature of the study, quantitative data regarding blood flow during dialysis sessions was not available for many patients. Lastly, because this study focused on an ICU cohort, the results cannot be generalized to patient populations in other healthcare settings. Despite these limitations, the longitudinal comparison provides a comprehensive assessment of these catheters’ clinical performance, underscoring the VectorFlow catheter’s potential to improve treatment outcomes through its spiral laminar flow design.

## Conclusion

In conclusion, the study’s findings highlight the significant advancements in catheter technology, as evidenced by the VectorFlow catheter’s superior performance in terms of primary unassisted patency. These findings suggest that catheter tip design has a significant impact on clinical outcomes and support a VectorFlow-first placement strategy within this critical patient population.

## Data Availability

Data will be made available on reasonable request.
